# Comparison of APACHE IV with APACHE II, SAPS 3, MELD, MELD-Na, and CTP scores in predicting mortality after liver transplantation

**DOI:** 10.1038/s41598-017-07797-2

**Published:** 2017-09-07

**Authors:** Hannah Lee, Susie Yoon, Seung-Young Oh, Jungho Shin, Jeongsoo Kim, Chul-Woo Jung, Ho Geol Ryu

**Affiliations:** 1 Departments of Anesthesiology and Pain Medicine, Seoul National University College of Medicine, Seoul National University Hospital, Seoul, Korea; 2 Departments of Surgery, Seoul National University College of Medicine, Seoul National University Hospital, Seoul, Korea

## Abstract

The Acute Physiology and Chronic Health Evaluation (APACHE) IV score and Simplified Acute Physiology Score (SAPS) 3 include liver transplantation as a diagnostic category. The performance of APACHE IV-liver transplantation (LT) specific predicted mortality, SAPS 3, APACHE II, Model for End-stage Liver Disease (MELD)-Na, MELD, and CTP scores in predicting in-hospital and 1 year mortality in liver transplant patients was compared using 590 liver transplantations in a single university hospital. In-hospital mortality and 1 year mortality were 2.9% and 4.2%, respectively. The APACHE IV-LT specific predicted mortality showed better performance in predicting in-hospital mortality (AUC 0.91, 95% CI [0.86–0.96]) compared to SAPS 3 (AUC 0.78, 95% CI [0.66–0.90], *p* = 0.01), MELD-Na (AUC 0.74, 95% CI [0.57–0.86], *p* = 0.01), and CTP (AUC 0.68, 95% CI [0.54–0.81], *p* = 0.01). The APACHE IV-LT specific predicted mortality showed better performance in predicting 1 year mortality (AUC 0.83, 95% CI [0.76–0.9]) compared to MELD-Na (AUC 0.67, 95% CI [0.55–0.79], *p* = 0.04) and CTP (AUC 0.64, 95% CI [0.53–0.75], *p* = 0.03), and also in all MELD groups and in both living and deceased donor transplantation. The APACHE IV-LT specific predicted mortality showed better performance in predicting in-hospital and 1 year mortality after liver transplantation.

## Introduction

Liver transplantation has become the standard treatment for irreversible acute liver failure and end-stage liver diseases. Advances in surgical technique and post-operative care have markedly decreased early mortality rate after liver transplantation^1, 2^. Nevertheless, given the relatively high risk of surgery and limited availability of organs, predicting the short-term outcome of liver transplant recipients using various scores and models continues to be important^3, 4^.

The Model for End-stage Liver Disease (MELD) score, developed in the late 1990s^5^, has been incorporated into the organ allocation system since 2002^6^. The correlation between preoperative MELD scores and early mortality has been studied with mixed results^3, 4, 7^. Acute Physiology and Chronic Health Evaluation (APACHE) scores and Simplified Acute Physiology Score (SAPS) models are widely used for severity of illness assessment and outcome predictions in critically ill patients^8, 9^. Studies comparing MELD scores with APACHE II in liver transplant patients^10^ and APACHE II with SAPS 3 scores in solid organ transplant patients^11^ have shown inconclusive results.

Liver transplantation was not incorporated into scores or models until the recently updated APACHE IV and SAPS 3 models^12, 13^. However, there has been no comparison of APACHE IV and SAPS 3 with other outcome prediction models in patients undergoing liver transplantation. Therefore, we compared the performance of prognostic models in predicting early mortality in liver transplant patients: APACHE IV-LT specific predicted mortality, SAPS 3, APACHE II, MELD-Na, MELD, and Child-Turcotte-Pugh (CTP) scores. Factors associated with in-hospital mortality after liver transplantation were also evaluated.

## Results

### Characteristics of the study population

Between October 2010 and September 2014, 633 patients who had undergone living donor or deceased donor liver transplantation were admitted to the surgical ICU. After excluding 42 pediatric patients and one re-transplantation patient, 590 patients were included for analysis. MELD scores were < 15 in 309 (52.4%) patients, 15 to 24 in 161 (27.3%) patients, and ≥25 in 120 (20.7%) patients. Seventeen of the 590 patients (2.9%) died in the hospital after liver transplantation. Causes of in-hospital mortality included sepsis (8 patients), postoperative massive bleeding (3 patients), primary allograft nonfunction (2 patients), acute respiratory distress syndrome due to pneumonia (2 patients), massive pulmonary thromboembolism (1 patient), and brain herniation (1 patient). Including the 8 patients who were discharged but died within 1 year after liver transplantation, the overall 1 year mortality was 4.2% (25/590).

### Comparison among models in predicting in-hospital mortality

**Figure 1:**
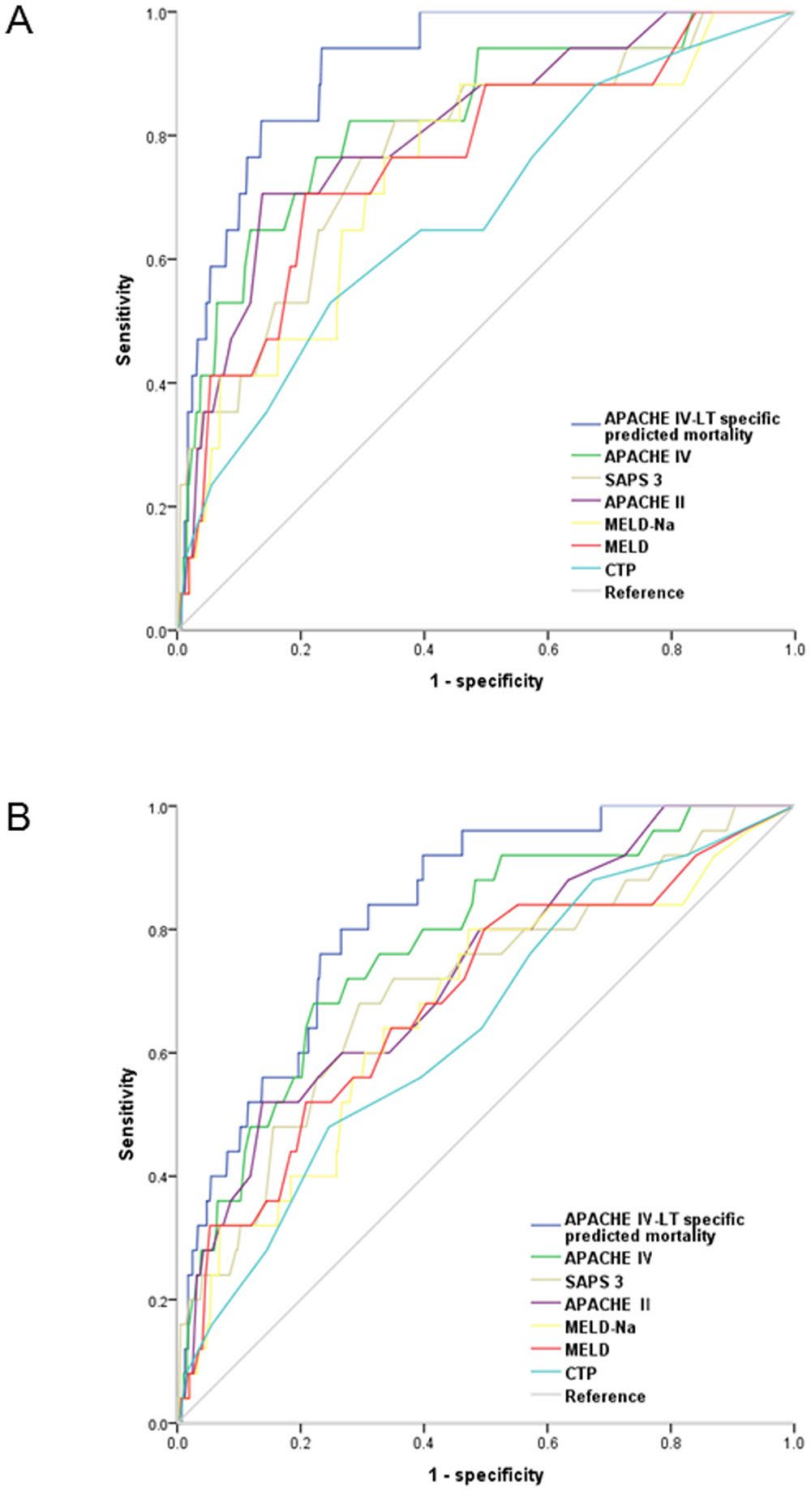
Comparison of the ROC curves of APACHE IV-liver transplantation specific predicted mortality, APACHE IV, SAPS 3, APACHE II MELD-Na, MELD, and CTP scores in predicting in-hospital (**A**) and 1 year mortality (**B**). (**A**) The AUCs are 0.91, 0.83, 0.78, 0.81, 0.74, 0.76, and 0.68 in APACHE IV-liver transplantation specific predicted mortality, APACHE IV, SAPS 3, APACHE II, MELD-Na, MELD, and CTP models, respectively. (**B**) The AUCs are 0.83, 0.78, 0.71, 0.73, 0.67, 0.69, and 0.64 in APACHE IV-liver transplantation specific predicted mortality, APACHE IV, SAPS 3, APACHE II, MELD-Na, MELD, and CTP models, respectively. ROC, receiver operating characteristic; APACHE, acute physiology and chronic health evaluation; SAPS, Simplified Acute Physiology Score; CTP, Child-Turcotte-Pugh score.

APACHE IV-LT specific predicted mortality showed excellent discrimination in predicting in-hospital mortality with an AUC of 0.91 (95% CI [0.86–0.96]) (Table 1). After adjusting for multiple comparison using the Holm method, APACHE IV-LT specific predicted mortality showed larger AUCs compared to SAPS 3, MELD-Na, and CTP (Table 1). Discrimination was very good or good for all other models in predicting in-hospital mortality except for CTP score (Fig. 1A). All 6 prognostic models showed good calibration and adequately described the in-hospital mortality pattern (Table 1, Supplementary Fig. 1). The APACHE IV score also showed very good discrimination in predicting in-hospital mortality with an AUC of 0.83 (95% CI [0.72–0.94]).

**Table 1:** Performance of APACHE IV, SAPS 3, APACHE II, MELD-Na, MELD, and CTP models on prediction of in-hospital mortality.

	APACHE IV -LT specific predicted mortality	SAPS 3	APACHE II	MELD-Na	MELD	CTP
AUC (95% CI)	0.91 (0.86–0.96)	0.78 (0.66–0.90)^*^	0.81 (0.70–0.92)	0.74 (0.57–0.86)^†^	0.76 (0.64–0.89)	0.68 (0.54–0.81)^‡^
Cutoff point	54	56	20	22	21	10
H-L C-test χ^2^	6.70	5.47	6.78	5.96	11.99	4.48
*p-*value	0.57	0.71	0.56	0.65	0.15	0.48
H-L H-test χ^2^	7.35	9.42	6.85	2.00	5.63	3.9
*p-*value	0.50	0.31	0.55	0.98	0.69	0.87
SMR (95% CI)	NA	0.13 (0.08–0.21)	0.10 (0.06–0.16)	NA	NA	NA
Sensitivity	0.83	0.76	0.71	0.76	0.71	0.65
Specificity	0.84	0.70	0.86	0.66	0.79	0.60
PPV	0.13	0.07	0.13	0.06	0.09	0.05
NPV	0.99	0.99	0.99	0.99	0.99	0.98

Statistical comparison of APACHE IV-LT specific predicted mortality with ^*^SAPS 3 scores (*p* = 0.012), ^†^MELD-Na scores (*p* = 0.012), and ^‡^CTP scores (*p* = 0.009) after Holm adjustment for multiple comparisons.

APACHE, acute physiology and chronic health evaluation; SAPS, Simplified Acute Physiology Score; LT, liver transplantation, MELD, model for end-stage liver disease; MELD-Na, model for end-stage liver disease-Na; CTP, Child-Turcotte-Pugh score; AUC, area under the receiver operating curve; CI, confidence interval; PPV, positive predictive value; NPV, negative predictive value; H-L C-test. Hosmer-Lemeshow C-statistics; H-L H-test, Hosmer-Lemeshow H-statistics; NA, not applicable; SMR, Standardized mortality ratio.

APACHE IV-LT specific predicted mortality showed excellent or very good discrimination in all MELD score groups with better performance compared to SAPS 3 or APACHE II in patients with MELD scores between 15 and 24, and CTP in patients with MELD scores less than 15 (Table 2). In deceased donor liver transplantation, APACHE IV-LT specific predicted mortality showed very good discrimination and better performance compared to SAPS 3 or MELD-Na scores (Table 2).

**Table 2:** Comparison of APACHE IV, SAPS 3, APACHE II, MELD-Na, and CTP scores according to MELD score in predicting in-hospital mortality.

MELD score or donor status	Non-survivor/total patients	AUC (95% confidence interval)					
		APACHE IV -LT specific predicted mortality	SAPS 3	APACHE II	MELD-Na	MELD	CTP
<15	4/309	0.93 (0.81–1.00)	0.82 (0.58–1.00)	0.77 (0.52–1.00)	0.62 (0.30–0.94)	—	0.57 (0.32–0.81)^*^
15–24	3/161	0.87 (0.75–0.98)	0.61 (0.39–0.84)^†^	0.52 (0.24–0.79)^‡^	0.65 (0.52–0.77)	—	0.59(0.22–0.96)
≥25	10/120	0.84 (0.53–1.00)	0.70 (0.52–0.89)	0.80 (0.69–0.91)	0.58 (0.37–0.79)	—	0.56 (0.35–0.77)
Living donor	6/412	0.91 (0.82–1.00)	0.72 (0.47–0.96)	0.80 (0.62–0.99)	0.61 (0.38–0.85)	0.65 (0.42–0.88)	0.62 (0.39–0.84)
Deceased donor	11/178	0.87 (0.82–0.92)	0.71 (0.55–0.86)^§^	0.73 (0.45–0.90)	0.73 (0.57–0.88)^¶^	0.76 (0.61–0.92)	0.61 (0.40–0.81)
All	17/590	0.91 (0.86–0.96)	0.78 (0.66–0.90)	0.81 (0.70–0.92)	0.74 (0.57–0.86)	0.76 (0.64–0.89)	0.68 (0.54–0.81)

Statistical comparison of APACHE IV-LT specific predicted mortality according to MELD scores with ^*^CTP scores (*p* < 0.001), ^†^SAPS 3 scores (*p* = 0.012), and ^‡^APACHE II scores (*p* < 0.001) after Holm adjustment for multiple comparisons.

Statistical comparison of APACHE IV-LT specific predicted mortality in deceased donor liver transplantation with ^§^SAPS 3 scores (*p* = 0^.^012) and ^¶^MELD-Na scores (*p* < 0^.^001) after Holm adjustment for multiple comparisons.

APACHE, acute physiology and chronic health evaluation; LT, liver transplantation; MELD, model for end-stage liver disease; MELD-Na, model for end-stage liver disease-Na; SAPS, Simplified Acute Physiology Score; CTP, Child-Turcotte-Pugh score.

### Factors associated with in-hospital mortality

Due to collinearity, donor status and MELD scores were chosen over operation type (Pearson’s correlation coefficient 0.950) and MELD-Na scores (Pearson’s correlation coefficient 0.797) for univariable and multivariable analyses, respectively.

Compared to in-hospital survivors, non-survivors had higher MELD, MELD-Na, and CTP scores before transplantation and higher APACHE IV, SAPS 3, and APACHE II scores (Table 3). Non-survivors were more likely to require vasopressor support at ICU admission, require more postoperative transfusion, develop AKI, and require preoperative and postoperative renal replacement therapy (Table 3).

**Table 3:** Patient characteristics of in-hospital survivors and non-survivors.

Variables	In-hospital survivor (n = 573)	In-hospital non-survivor (n = 17)	*p-* value
Age (years)	54 [9]	58 [13]	0.227*
Sex (M/F)	405 (70.7)/168 (29.3)	13 (76.5)/4 (23.5)	0.789
Body mass index (kg/m^2^)	24.5 [12.1]	25.0 [2.7]	0.832*
Initial Diagnosis			
Hepatocellular carcinoma	317 (55.3)	8 (47.1)	0.622
Liver cirrhosis			
Hepatitis B virus liver cirrhosis	379 (66.1)	9 (52.9)	0.302
Hepatitis C virus liver cirrhosis	57 (9.9)	3 (17.6)	0.402
Alcoholic liver cirrhosis	76 (13.3)	3 (17.6)	0.487
Others	35 (6.1)	1 (5.9)	1.000
Preoperative sodium (mmol/L)	135 [7]	138 [7]	0.071
Preoperative corrected sodium (mmol/L)	135 [7]	138 [7]	0.073
MELD score	16 [9]	27 [12]	<0.001*
MELD-Na score	18 [10]	28 [11]	0.002*
CTP score	8 [3]	10 [3]	0.009*
Coexisting conditions			
Diabetes mellitus	134 (23.4)	4 (23.5)	1.000
Hypertension	97 (16.9)	3 (17.6)	1.000
Chronic kidney disease	28 (4.9)	1 (5.9)	0.581
Preoperative RRT	19 (3.3)	3 (17.6)	0.022
*Perioperative factors*			
Donor status			
Living/Deceased	406 (70.9)/ 167 (29.1)	6 (35.3)/ 11 (64.7)	0.005
Operation type			
Elective/ Emergency	396 (69.1)/ 177 (30.9)	5 (29.4)/ 12 (70.6)	0.001
Operation time (min)	390 [93]	374 [86]	0.493*
Intraoperative RBC (units)	7 [9]	8 [4]	0.545*
*Postoperative factors*			
APACHE IV score	66 [21]	97 [26]	<0.001*
APACHE IV-LT specific predicted mortality (%)	29.2 [23.3]	73.3 [19.3]	<0.001*
SAPS 3 score	48 [14]	65 [18]	<0.001*
SAPS 3 predicted mortality (%)	19.9 [19.3]	44.9 [28.2]	0.003*
APACHE II score	17 [7]	26 [8]	<0.001*
APACHE II predicted mortality (%)	28.1 [16.0]	55.4 [23.9]	<0.001*
APACHE II predicted mortality- LT specific diagnostic weight (%)	14.3 [13.8]	34.5 [22.7]	0.001*
Inotropic support on admission to ICU	32 (5.6)	5 (29.4)	0.003
Mechanical ventilation duration (hours)	17 [45]	220 [306]	0.015*
Postoperative AKI	77 (13.5)	13 (76.5)	<0.001
Postoperative RRT	19 (3.3)	10 (58.8)	<0.001
Biliary complications	55 (9.6)	2 (11.8)	0.675
Reoperation	65 (11.4)	4 (23.5)	0.127
Postoperative RBC (units)	2 [7]	10 [9]	0.001*
Surgical site infection	29 (5.1)	6 (35.3)	<0.001
ICU readmission	27 (4.7)	7 (41.2)	<0.001
Postoperative ICU LOS (days)	5.6 [5.5]	39.0 [61.9]	0.041*
Hospital LOS (days)	30 [25]	60 [59]	0.046*
Preoperative hospital LOS (days)	10 [11]	20 [23]	0.077*
Postoperative hospital LOS (days)	20 [19]	40 [61]	0.151*

Data are expressed as mean [standard deviation] or number (%). *Mann-Whitney U test.

MELD, model for end-stage liver disease; MELD-Na, model for end-stage liver disease-Na; APACHE, acute physiology and chronic health evaluation; LT, liver transplantation; SAPS, Simplified Acute Physiology Score; CTP, Child-Turcotte-Pugh score; AKI, acute kidney injury; RRT, renal replacement therapy; ICU, intensive care unit; LOS, length of stay.

After adjusting for relevant factors with *p* < 0.2 in univariable analyses, APACHE IV-LT specific predicted mortality, preoperative corrected sodium level, preoperative RRT, postoperative RRT, and ICU readmission were identified as independent factors associated with in-hospital mortality (Table 4). After adjusting for variables with *p* < 0.1 in the univariable analyses, APACHE IV-LT specific predicted mortality (OR 1.06, 95% CI [1.03–1.10], *p* < 0.001), preoperative corrected sodium level (OR 1.12, 95% CI [1.00–1.26], *p* = 0.05), postoperative RRT (OR 16.75, 95% CI [4.37–64.16], *p* < 0.001), and ICU readmission (OR 8.33 [1.83–38.05], *p* = 0.01) were identified as independent factors associated with in-hospital mortality (Supplementary Table 1). In living donor liver transplantation, ICU readmission (OR 54.83, 95% CI [2.79–1076.08], *p* = 0.008) and inotropic support on admission to ICU (OR 28.76, 95% CI [1.14–725.46], *p* = 0.041) were independent risk factors of in-hospital mortality (Supplementary Table 2), whereas preoperative corrected sodium levels (OR 1.17. 95% CI [1.01–1.35], *p = *0.036) and preoperative RRT (OR 17.72, 95%CI [1.51–208.36], *p* = 0.022) were independent risk factors in deceased donor liver transplantation (Supplementary Table 3).

**Table 4:** Factors associated with in-hospital mortality after liver transplantation.

Variables	Unadjusted OR (95% CI)	*P-* value in univariable- analysis	Adjusted OR^†^ (95% CI)	*P-* value in multivariable- analysis
Age	1.053 (0.994–1.116)	0.079		
Gender (female)	0.742 (0.238–2.308)	0.606		
Body mass index (kg/m^2^)	1.003 (0.974–1.033)	0.834		
Initial Diagnosis				
Hepatocellular carcinoma	0.718 (0.273–1.887)	0.501		
Liver cirrhosis				
Hepatitis B virus LC	0.576 (0.219–1.516)	0.264		
Hepatitis C virus LC	1.940 (0.541–6.953)	0.309		
Alcoholic LC	1.401 (0.393–4.990)	0.603		
Others	0.961 (0.124–7.455)	0.969		
Preoperative corrected sodium	1.087 (0.992–1.191)	0.073	1.142 (1.013–1.287)	0.029
MELD score	1.090 (1.046–1.136)	<0.001		
Coexisting conditions				
Diabetes	1.008 (0.323–3.143)	0.989		
Hypertension	1.052 (0.297–3.729)	0.938		
Chronic kidney disease	1.217 (0.256–9.504)	0.852		
Preoperative RRT	6.248 (1.656–23.581)	0.007	6.962 (1.154–42.004)	0.025
*Perioperative factors*				
Donor status (deceased)	4.457 (1.622–12.248)	0.004		
Recipient operation time (min)	0.998 (0.993–1.004)	0.492		
Intraoperative RBC (units)	1.015 (0.967–1.065)	0.229		
*Postoperative factor*				
APACHE IV-LT specific predicted mortality*	1.069 (1.043–1.096)	<0.001	1.062 (1.033–1.093)	<0.001
Inotropic support on admission to ICU	7.285 (2.415–21.979)	0.001		
Postoperative AKI	20.935 (6.655–65.858)	<0.001		
Postoperative RRT	45.757 (14.230–147.127)	<0.001	17.544 (4.778–64.418)	<0.001
Biliary complication	1.253 (0.279–5.625)	0.768		
Reoperation	2.400 (0.760–7.580)	0.136		
Postoperative RBC (units)	1.041 (1.008–1.076)	<0.001		
Surgical site infection	10.213 (3.529–29.554)	<0.001		
ICU readmission	10.617 (3.661–30.790)	<0.001	8.070 (1.700–38.301)	0.009
Preoperative hospital LOS	1.043 (1.017–1.070)	0.001		

*p-*value of Hosmer-Lemeshow goodness-of-fit test of multivariable analysis: 0.982. Nigelkerke R^2:^ 0.604.

*APACHE IV-LT specific predicted mortality which has the highest AUC was chosen as a representative variable among other scoring systems for multivariable analysis.

^†^After adjusting for MELD score, donor status, vasopressors on admission, reoperation, postoperative RBC transfusion, surgical site infection, and preoperative hospital stay.

LC, liver cirrhosis; MELD, model for end-stage liver disease; MELD-Na, model for end-stage liver disease-Na; APACHE, acute physiology and chronic health evaluation; SAPS, Simplified Acute Physiology Score; CTP, Child-Turcotte-Pugh score; AKI, acute kidney injury; RRT, renal replacement therapy; ICU, intensive care unit; LOS, length of stay.

### Comparison among models in predicting 3-month mortality

APACHE IV-LT specific predicted mortality showed very good discrimination in predicting 3-month mortality with an AUC of 0.87 (95% CI [0.79–0.95]) (Supplementary Table 4). After adjusting for multiple comparison using the Holm method, APACHE IV-LT specific predicted mortality showed larger AUCs compared to CTP (*p* = 0.02, Supplementary Table 4). Discrimination was very good or good for all other models in predicting 3-month mortality except for CTP score (Supplementary Table 4). All 6 prognostic models showed good calibration and adequately described the 3-month mortality pattern (Supplementary Table 4).

### Comparison among models in predicting 1 year mortality

In predicting 1 year mortality, APACHE IV-LT specific predicted mortality showed an AUC of 0.83 (95% CI [0.76–0.90]), indicating very good discrimination (Fig. 1B) and all 6 models showed good calibration (Table 5, Supplementary Fig. 2). After adjusting for multiple comparison using the Holm method, the AUC of APACHE IV-LT specific predicted mortality was larger compared to MELD-Na (*p* = 0.035) and CTP (*p * = 0.030) (Table 5).

**Table 5:** Performance of APACHE IV, SAPS 3, APACHE II, MELD-Na, MELD, and CTP models on prediction of 1 year mortality.

	APACHE IV -LT specific predicted mortality	SAPS 3	APACHE II	MELD-Na	MELD	CTP
AUC (95% CI)	0.83 (0.76–0.90)	0.71 (0.59–0.82)	0.73 (0.63–0.83)	0.67 (0.55–0.79)^*^	0.69 (0.57–0.80)	0.64 (0.53–0.75) ^†^
Cutoff point	39	55	20	23	19	11
H-L C-test χ^2^	7.91	2.81	6.75	8.46	6.73	4.37
*p-*value	0.44	0.95	0.56	0.39	0.57	0.50
H-L H-test χ^2^	5.92	6.53	8.62	6.37	8.92	5.19
*p-*value	0.66	0.59	0.38	0.61	0.35	0.74
SMR (95% CI)	NA	0.19 (0.13–0.29)	0.15 (0.09–0.22)	NA	NA	NA
Sensitivity	0.72	0.68	0.60	0.64	0.64	0.48
Specificity	0.73	0.70	0.73	0.67	0.65	0.75
PPV	0.11	0.09	0.09	0.08	0.08	0.08
NPV	0.98	0.98	0.98	0.98	0.98	0.97

Statistical comparison of APACHE IV-LT specific predicted mortality with *MELD-Na scores (*p* = 0.035) and ^†^CTP scores (*p* = 0.03) after Holm adjustment for multiple comparisons.

APACHE, acute physiology and chronic health evaluation; LT, liver transplantation; MELD, model for end-stage liver disease; MELD-Na, model for end-stage liver disease-Na; SAPS, Simplified Acute Physiology Score; CTP, Child-Turcotte-Pugh score; AUC, area under the receiver operating curve; CI, confidence interval; PPV, positive predictive value; NPV, negative predictive value; H-L C-test. Hosmer-Lemeshow C-statistics; H-L H-test, Hosmer-Lemeshow H-statistics; SMR, Standardized mortality ratio; NA, not applicable.

APACHE IV-LT specific predicted mortality showed very good or good discrimination in all MELD score groups but did not show any significant difference compared to other models (Supplementary Table 5) In deceased donor liver transplantation, APACHE IV-LT specific predicted mortality showed good discrimination and better performance compared to SAPS 3 (*p* < 0.001), APACHE II scores (*p* < 0.001), and MELD-Na scores (*p* = 0.002) (Supplementary Table 5).

Compared to 1 year survivors, non-survivors showed higher MELD, MELD-Na, and CTP scores before transplantation and higher APACHE IV, SAPS 3, and APACHE II scores (Supplementary Table 6). Non-survivors were more likely to require vasopressors at ICU admission, receive more intraoperative and postoperative transfusion, develop AKI, require longer duration of mechanical ventilation, and require preoperative and postoperative renal replacement therapy (Supplementary Table 6).

### Comparison of mortality by subgroups

Between groups of patients with MELD scores < 15 and MELD scores ≥ 25, there was a 5.2% to 8.6% difference in survival rate for up to 18 months after transplantation (Supplementary Table 7, Supplementary Fig. 3). Living donor liver transplant patients had higher survival rates compared to deceased donor liver transplant patients (1.5% vs 6.2%, *p* = 0.005). Lower APACHE IV scores correlated with higher survival rates (Supplementary Table 8, Supplementary Fig. 4).

## Discussion

The main findings of this study are that the APACHE IV-LT specific predicted mortality 1) showed very good to excellent discrimination and calibration in predicting in-hospital and 1 year mortality after liver transplantation, 2) showed better discrimination in in-hospital and 1 year mortality compared to other scores, and 3) was the only model that showed good to excellent discrimination in in-hospital and 1 year mortality in all MELD groups and in both living and deceased donor liver transplantation.

The APACHE II score^8^, introduced in 1985, is an old version of the APACHE system but still widely used because of its simplicity and capability of classifying severity of illness and predicting hospital mortality^14^. The APACHE II score did not have liver transplantation in the diagnostic category and was shown to overestimate in-hospital mortality in postoperative liver transplantation patients unless orthotopic liver transplantation specific diagnostic weight was applied^15^. The liver transplant-specific coefficients using original APACHE II score was reported to be a good predictor of hospital and 1 year mortality after liver transplantation^16^ and in our study, the performance of liver transplant-specific coefficient of APACHE II score was similar to the performance of the APACHE II score. The APACHE IV score was developed in 2006 and has been widely implemented to general ICUs and specific patient groups^12, 17, 18^. A major advantage of the APACHE IV model is its accommodation of 116 detailed admitting diagnostic options, including postoperative liver transplantation, which promotes outcome analysis in specific subgroups^12^. A recent study of 195 orthotopic liver transplant patients showed that APACHE IV score (AUC 0.94) demonstrated better performance compared to MELD score (AUC 0.69) in predicting in-hospital mortality after deceased donor liver transplantation^19^. Despite the discrepancy between our study population and that from which liver transplant-specific diagnostic weighted equation of APACHE IV for mortality prediction was derived (70% living donor liver transplantation vs. 158 orthotopic liver transplantation only)^12^, our results were similar and showed that APACHE IV outperformed other scores.

Since the development of MELD scores in 2000 to predict 3-month mortality after transjugular intrahepatic portosystemic shunt (TIPS)^5^, MELD scores have been used to prioritize liver allocation and predict mortality of liver cirrhosis patients awaiting liver transplantation^20^. However, MELD scores that incorporated sodium (MELD-Na) were shown to better predict mortality among candidates for liver transplantation compared with the MELD score^21^. Consequently, serum sodium was recently added to the MELD score by the OPTN. The original MELD score and the MELD-Na were included in our study for comparison with other scoring systems. Similar to SAPS 3, the discrimination and calibration of MELD and MELD-Na scores were good in predicting in-hospital and 3-month mortality. However, the APACHE IV-LT specific predicted mortality showed better discrimination in predicting 1 year mortality compared to MELD-Na scores. A previous study has also shown similar results^20^ and may be attributed to the original purpose of the scores and that only values prior to liver transplantation are incorporated.

The SAPS 3 model was developed in 2005^22^ and has shown good discrimination in ICU patients^18, 23^. The SAPS 3 model also has subgroups of admission categories including the anatomical site of surgery. Transplantation- specific diagnostic weighted equation was derived from 172 transplant patients, 90 of which were liver transplantations^13^. In 152 orthotopic liver transplant patients, SAPS 3 was similar to APACHE II in predicting in-hospital mortality after liver transplantation with moderate discrimination^11^. Similarly, the performance of SAPS 3 in our study in predicting in-hospital and 1 year mortality was comparable to other models, except for APACHE IV-LT specific predicted mortality.

The CTP score has been used as a classic tool to grade the severity of liver disease^24^. Previous studies comparing CTP with MELD and APACHE II scores suggest that the CTP score is less accurate in predicting early and late post-transplant mortality^10, 25^. The lack of extrahepatic parameters and physiologic variables and the basis on which the CTP score was developed may account for its poor discriminative performance in predicting in-hospital mortality and 1 year mortality after liver transplantation, as shown again in our study.

When comparing different scoring systems, differences in incorporated variables, study population or patient mix, time between development of the model and patient enrollment, mortality rates, and sample size between the study population and the original cohort used in the development of the scoring system should be considered^17, 26^. More specifically, APACHE and SAPS scores are calculated after ICU admission and incorporate comorbidities, postoperative vital signs, and laboratory values with an aim to predict in-hospital mortality, whereas MELD and CTP scores only account for select preoperative values, mostly related to hepatic function, with an aim to assess the severity of liver dysfunction. Consequently, the performance of APACHE IV, SAPS 3, and APACHE II tend to be better compared to MELD-Na, MELD and CTP scores in predicting in-hospital mortality and 1 year mortality. In addition, unique perioperative aspects of hepatic dysfunction and liver transplantation such as hypotension, lactic acidosis, and coagulopathy followed by subsequent rapid recovery after transplantation may be reflected in APACHE scores and SAPS^27, 28^. Liver transplant patients are unique in that the wide variety of abnormalities quickly recover after transplantation, which may explain the inaccuracy of APACHE II when the diagnostic category weight of ‘postoperative gastrointestinal surgery’ is used^15^.

APACHE IV-LT specific predicted mortality showed excellent or very good discrimination in all MELD score groups and outperformed other models in predicting in-hospital mortality. APACHE IV was also the only scoring system that showed good or better discrimination in living donor and deceased donor liver transplantation. The APACHE IV- post liver transplant specific weighted equation that contains detailed postoperative vital signs and laboratory values may explain the superior performance in all aspects compared to other scores.

In accordance with our study, ICU readmission has been known to be highly correlated with in-hospital mortality not only in general ICU population but also in liver transplant patients^29, 30^. In our study, 41.2% of non-survivors were readmitted to the ICU after initial ICU discharge within the same hospital stay whereas only 4.7% of survivors were readmitted. Frequent causes of ICU readmission include postoperative bleeding, respiratory complications, and sepsis. Renal dysfunction is common in patients awaiting liver transplantation and after liver transplantation and has significant impact on perioperative and long-term morbidity and mortality^31^. In patients awaiting liver transplantation, the predicted 3-month mortality rate in patients on dialysis is up to 10 times higher compared to patients who do not require dialysis^5, 21^.

There are a few limitations to our study. Our study was conducted in a single center with a high proportion of living donor liver transplantation and hepatitis B patients. Similar to our study results, deceased donor liver transplantation have been associated with worse outcome compared to living donor liver transplantation^32^. The superior performance of the APACHE IV score in our study is most prominent in deceased donor liver transplant patients who have more severe preoperative conditions. Therefore, our results should be interpreted and applied taking into account that the majority of our study population were living donor liver transplant patients with less severe preoperative conditions. Second, the in-hospital and 1 year mortality rate was less than 5%. The small proportion of non-survivors limits the assessment of predictive model performance. However, considering that most patients are monitored in the ICU after liver transplantation, validation of the APACHE IV score with 590 patients helps confirm the utility of the APACHE IV score in liver transplant patients. Third, identified risk factors of in-hospital and 1 year mortality such as preoperative and postoperative renal replacement therapy and ICU readmission showed relatively wide confidence intervals, which may be due to the small number of non-survivors. Therefore, application of these risk factors into different circumstances and patient mix should be done with caution.

In conclusion, the APACHE IV score showed good discrimination and calibration in predicting in-hospital and 1 year mortality after liver transplantation and in all MELD groups and in both living and deceased donor liver transplantation.

## Methods

This study was approved by the institutional review board of the Seoul National University Hospital (1506–096–681). Informed consent was waived by the IRB due to the retrospective design of the study.

### Patient population

Patients who had undergone living or deceased donor liver transplantation from October 2010 to September 2014 at Seoul National University Hospital were included in this study. Pediatric patients (<18 years of age) and re-transplantation patients were excluded.

### Data collection

Data were obtained from the electronic medical record database to calculate APACHE IV-LT specific predicted mortality, SAPS 3, APACHE II, MELD-Na, MELD, and CTP scores. Coexisting diseases, body mass index, preoperative Na, recipient operation time, donor status, operation type, numbers of intra- and postoperative RBC transfusion units, postoperative acute kidney injury and renal replacement therapy, reoperation, biliary complications, surgical site infections, ICU and hospital length of stay, in-hospital and 1 year mortality were recorded.

### Score calculation

APACHE IV-LT specific predicted mortality and APACHE II scores were calculated using the worst lab values obtained within 24 hours of ICU admission and SAPS 3 were calculated using the worst lab values within 1 hour of ICU admission. MELD and MELD-Na scores were calculated using the most recent pre-transplantation labs obtained in the 48 hours prior to liver transplantation. MELD-Na score incorporated by the Organ Procurement and Transplantation Network (OPTN) as of January 2016 (https://optn.transplant.hrsa.gov/news/meld-serum-sodium-policy-changes). CTP score was calculated using the most recent laboratory values and physical findings before transplantation^24^.

### Discrimination and calibration of prognostic models

Discrimination refers to the ability to rank patients correctly according to their risk of death and was assessed using the area under the receiver operating characteristic (ROC) curves (AUC)^33^. It was classified as excellent, very good, good, moderate, and poor when AUCs were 0.9 to 0.99, 0.8 to 0.89, 0.7 to 0.79, 0.6 to 0.69, or <0.6, respectively^33^. If a statistical significance was observed in the AUC curve, Youden index (max [sensitivity + specificity − 1]) was used to determine the optimal cut-off point for each score^34^. To further assess discrimination of each prognostic model, patients were stratified into 3 groups according to their MELD score: <15, 15 to 24, and ≥25, which largely correlate with former United Network for Organ Sharing (UNOS) statuses 3, 2B, and sick 2B and 2 A, respectively^7^ and by the type of donor (living vs deceased).

Calibration was defined as the ability of a model to describe the mortality pattern in the data. The Hosmer–Lemeshow goodness-of-fit test was used to evaluate the agreement between observed and expected number of survivors and non-survivors across all strata with equal number of patients (C statistics) or with 10 groups divided by expected mortality intervals (H statistics), with a non-significant *p*-value (>0.05) indicating good calibration^35^.

### Clinical outcomes

In-hospital, 3-month, and 1 year mortality were recorded. Postoperative acute kidney injury (AKI) was classified into risk, injury, and failure according to the risk, injury, failure, loss of kidney function, and end stage kidney disease criteria. Preoperative renal replacement therapy (RRT) was defined as RRT initiated before liver transplantation and continued thereafter. Postoperative RRT was defined as RRT that was applied only after liver transplantation.

### Statistical analysis

Data were reported as the mean [standard deviation] and percentages for qualitative variables. All variables were tested for normal distribution with the Shapiro-Wilk test. Student’s *t*-test was used for normally distributed continuous variables. Variables with non-normal distribution and sample size less than 30 were analysed by the Mann-Whitney U test. Chi-square test or Fisher’s exact test (if cell size ≤ 5) was used for categorical variables. *P* values < 0.05 were considered statistically significant.

The Delong method^36^ was used to measure and compare AUCs to assess discrimination for in-hospital and 1 year mortality. The Holm–Bonferroni correction for multiple comparisons was applied to control the family-wise error rate and minimize type I and type II errors^37^. Calibration was assessed using the Hosmer-Lemeshow goodness-of-fit C and H statistics, with a *P* value greater than 0.05 indicating good calibration^35^. The standardized mortality ratio was calculated by dividing the observed mortality rate by the predicted mortality rate.

To identify risk factors of in-hospital mortality after liver transplantation, univariable logistic regression was performed after determining differences between survivors and non-survivors using the t-test and chi-square test (two-tailed). Risk factors with *p* values < 0.2 and *p* values < 0.1 in the univariable analysis were entered into multivariable logistic regression with forward selection. Collinearity between variables was tested before modeling, and if present (Pearson’s correlation coefficient > 0.7), only one variable was entered into the statistical analysis. Patient survival was also analyzed according to the MELD score groups (<15, 15–24, and ≥25) and donor type (living donor/ deceased donor). Statistical analysis was performed with SAS (SAS system for Windows, version 9.3; SAS institute, Cary, NC) and R (version 3.2.1) statistical software.

## Electronic supplementary material

Supplementary figures and tables
