# Supplementary material for: Comparison of APACHE IV with APACHE II, SAPS 3, MELD, MELD-Na, and CTP scores in predicting mortality after liver transplantation
Source: Sci Rep. 2017 Sep 7;7:10884. doi: 10.1038/s41598-017-07797-2 (PMC5589917; doi:10.1038/s41598-017-07797-2)
Supplement: Supplementary file 1 — Supplementary figures and tables [file 41598_2017_7797_MOESM1_ESM.pdf]

Comparison of APACHE IV with APACHE II, SAPS 3, MELD, MELD-Na, and CTP scores in predicting mortality after liver transplantation

Hannah Lee, Susie Yoon, Seung-Young Oh, Jungho Shin, Jeongsoo Kim, Chul-Woo Jung, Ho Geol Ryu

Table of Contents

Supplementary Figure 1. Calibration plots of six different models in predicting in-hospital mortality.

X- axis represents an approximate decile patient of 10 groups. Y-axis represents mortality in percentage (left) and number of patients (right). The bars represent the number of patients. Open triangles represent mean predicted mortality and closed circles represent mean observed mortality.

Supplementary Figure 2. Calibration plots of six different models in predicting 1 year mortality.

X- axis represents an approximate decile patient of 10 groups. Y-axis represents mortality in percentage (left) and number of patients (right). The bars represent the number of patients. Open triangles represent mean predicted mortality and closed circles represent mean observed mortality.

Supplementary Figure 3. Kaplan-Meier plot for probability of survival according to MELD score groups.

Supplementary Figure 4. Kaplan-Meier plot for probability of survival according to APACHE IV score groups.

Supplementary Table 1. Factors associated with in-hospital mortality after liver transplantation.

(multivariable analysis with variables with univariable analysis $p < 0.1$)

Supplementary Table 2. Factors associated with in-hospital mortality after living donor liver transplantation.

Supplementary Table 3. Factors associated with in-hospital mortality after deceased donor liver transplantation

Supplementary Table 4. Performance of APACHE IV, SAPS 3, APACHE II, MELD-Na, MELD, and CTP models on prediction of 3-month mortality

Supplementary Table 5. Comparison of APACHE IV, SAPS 3, APACHE II, MELD-Na, and CTP scores according to MELD score in predicting 1-year mortality.

Supplementary Table 6. Comparison of patient characteristics between 1-year survivor and non-survivor.

Supplementary Table 7. Survival rate after liver transplantation according to MELD scores.

Supplementary Table 8. Survival rate after liver transplantation according to APACHE IV scores.

Supplementary Figure 1.

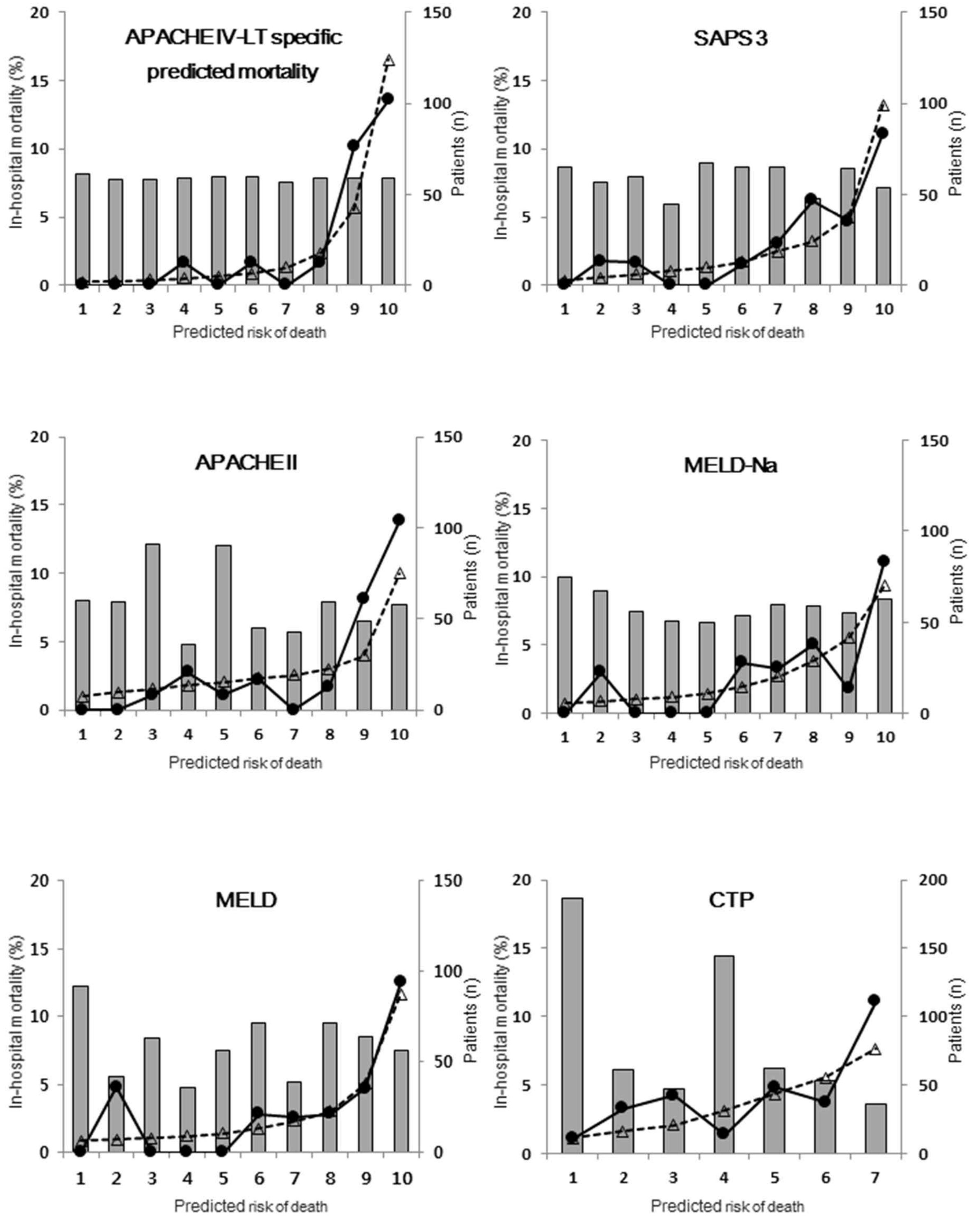

Supplementary Figure 2.

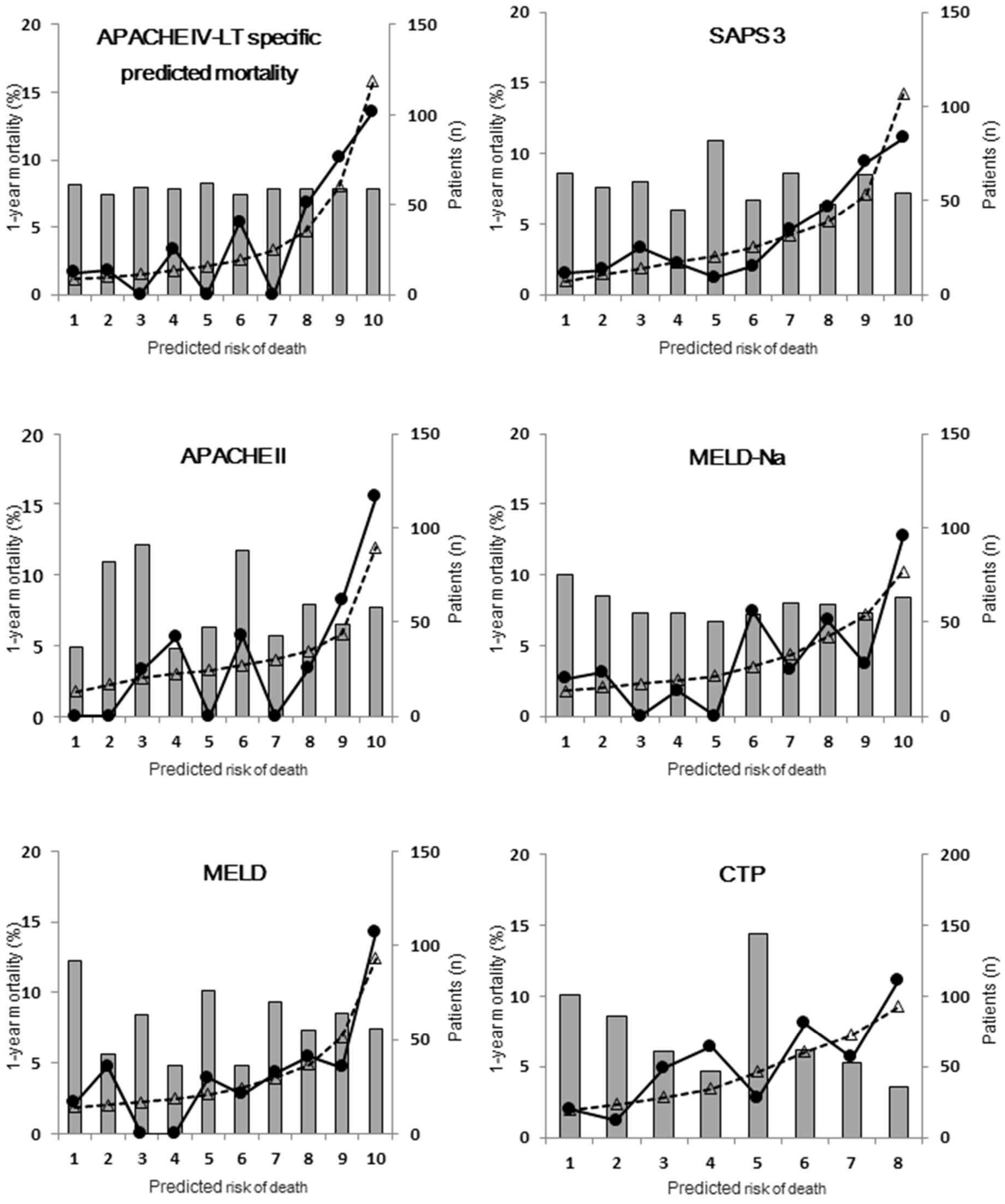

Supplementary Figure 3.

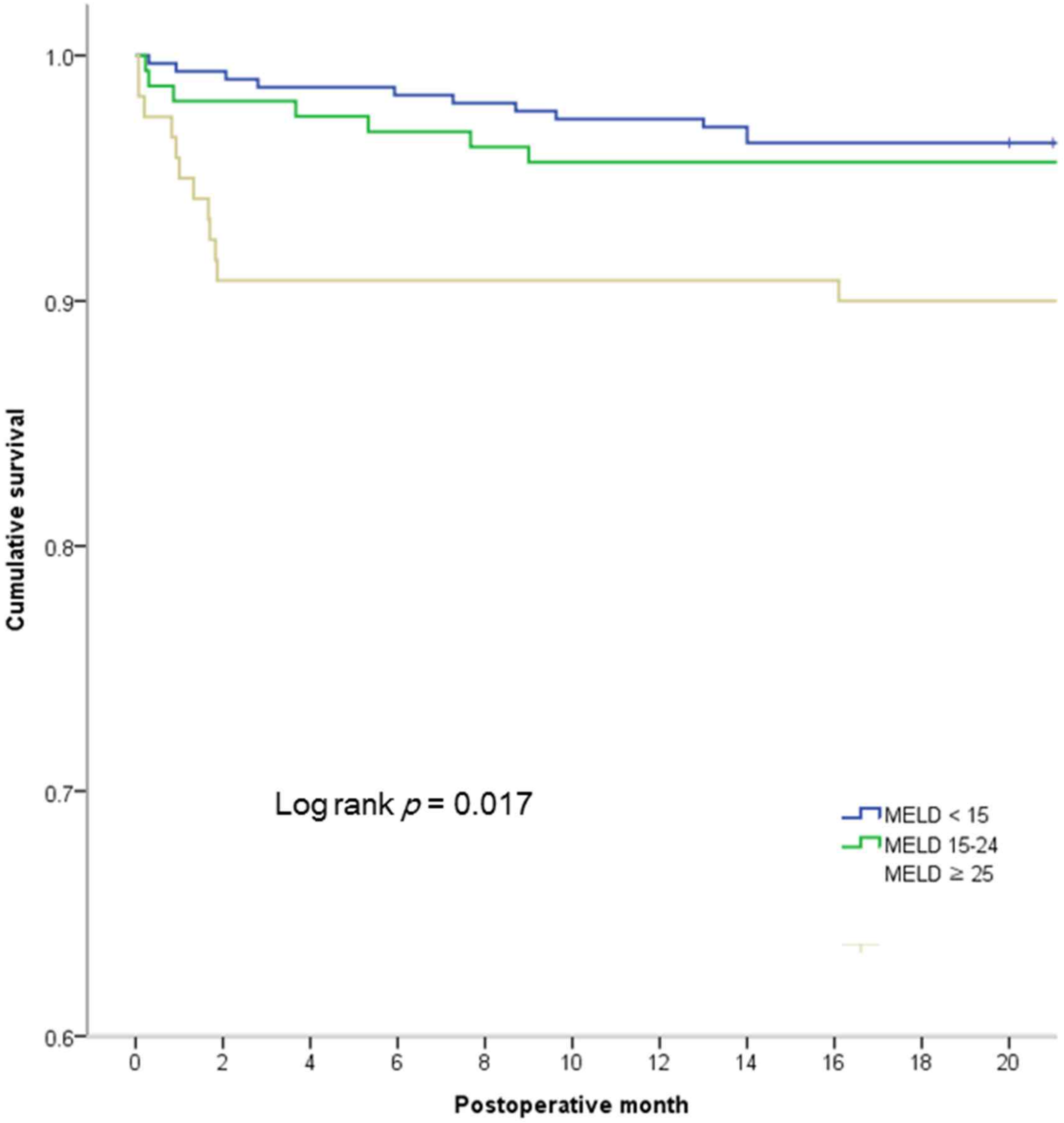

Month	0	1	3	6	12	18
Patient at risk (n)						
< 15	309	307	306	305	305	305
15-24	161	158	158	156	154	154
≥ 25	120	115	109	109	109	108

Supplementary Figure 4.

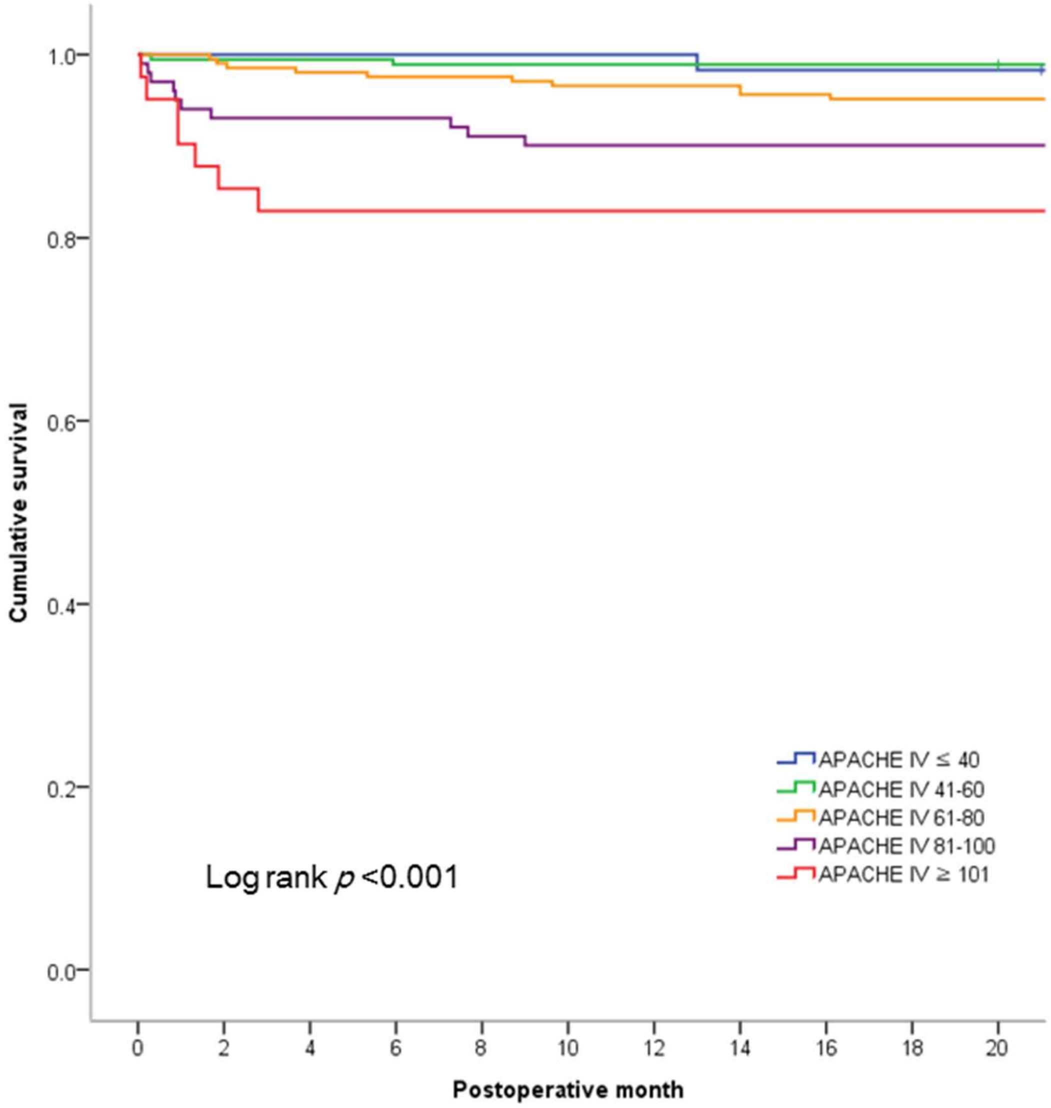

Month	0	1	3	6	12	18
Patient at risk						
≤ 40	59	59	59	59	59	59
41-60	183	182	182	182	182	182
61-80	206	206	204	204	203	203
81-100	101	56	55	55	55	55
≥ 101	41	37	34	34	34	34

Supplementary Table 1 Factors associated with in-hospital mortality after liver transplantation (multivariable analysis with variables with univariable analysis *p* <0.1)

Variables	Unadjusted OR (95% CI)	*P*- value in univariable-analysis	Adjusted OR† (95% CI)	*P*- value in multivariable-analysis
Age	1.053 (0.994-1.116)	0.079		
Gender (female)	0.742 (0.238-2.308)	0.606		
Body mass index (kg/m^2)	1.003 (0.974-1.033)	0.834		
Initial Diagnosis				
Hepatocellular carcinoma	0.718 (0.273-1.887)	0.501		
Liver cirrhosis				
Hepatitis B virus LC	0.576 (0.219-1.516)	0.264		
Hepatitis C virus LC	1.940 (0.541-6.953)	0.309		
Alcoholic LC	1.401 (0.393-4.990)	0.603		
Others	0.961 (0.124-7.455)	0.969		
Preoperative corrected sodium	1.087 (0.992-1.191)	0.073	1.124 (1.002-1.259)	0.045
MELD score	1.090 (1.046-1.136)	<0.001		
Coexisting conditions				
Diabetes	1.008 (0.323-3.143)	0.989		
Hypertension	1.052 (0.297-3.729)	0.938		
Chronic kidney disease	1.217 (0.256-9.504)	0.852		

Preoperative RRT	6.248 (1.656-23.581)	0.007		
Perioperative factors				
Donor status (deceased)	4.457 (1.622-12.248)	0.004		
Recipient operation time (min)	0.998 (0.993-1.004)	0.492		
Intraoperative RBC (units)	1.015 (0.967-1.065)	0.229		
Postoperative factor				
APACHE IV-LT specific predicted mortality *	1.069 (1.043-1.096)	<0.001	1.064 (1.034-1.095)	<0.001
Inotropic support on admission to ICU	7.285 (2.415-21.979)	0.001		
Postoperative AKI	20.935 (6.655-65.858)	<0.001		
Postoperative RRT	45.757 (14.230-147.127)	<0.001	16.747 (4.371-64.161)	<0.001
Biliary complication	1.253 (0.279-5.625)	0.768		
Reoperation	2.400 (0.760-7.580)	0.136		
Postoperative RBC (units)	1.041 (1.008-1.076)	<0.001		
Surgical site infection	10.213 (3.529-29.554)	<0.001		
ICU readmission	10.617 (3.661-30.790)	<0.001	8.333 (1.825-38.048)	0.006
Preoperative hospital LOS	1.043 (1.017-1.070)	0.001		

p- value of Hosmer-Lemeshow goodness-of –fit test of multivariable analysis: 0.988, Nigelkerke R^2:0.587.

*APACHE IV-LT specific predicted mortality which has the highest AUC was chosen as a representative variable among other scoring systems for multivariable analysis.

†After adjusting for MELD score, donor status, vasopressors on admission, reoperation, postoperative RBC transfusion, surgical site infection, and preoperative hospital stay.

LC, liver cirrhosis; MELD, model for end-stage liver disease; MELD-Na, model for end-stage liver disease-Na; APACHE, acute physiology and chronic health evaluation; SAPS, Simplified Acute Physiology Score; CTP,

Child-Turcotte-Pugh score; AKI, acute kidney injury; RRT, renal replacement therapy; ICU , intensive care unit; LOS, length of stay.

Supplementary Table 2 Factors associated with in-hospital mortality after living donor liver transplantation

Variables	Unadjusted OR (95% CI)	*P* value in univariable-analysis	Adjusted OR† (95% CI)	*P* value in multivariable - analysis
Age	1.122 (0.996-1.264)	0.057		
Body mass index (kg/m^2)	1.001 (0.951-1.054)	0.965		
Initial Diagnosis				
Hepatocellular carcinoma	1.160 (0.210-6.407)	0.865		
Liver cirrhosis				
Hepatitis B virus LC	0.420 (0.083-2.108)	0.292		
Hepatitis C virus LC	4.705 (0.835-26.518)	0.079		
Alcoholic LC	1.733 (0.198-15.191)	0.619		
Preoperative corrected sodium	1.189 (0.956-1.478)	0.119		
MELD score	1.048 (0.969-1.133)	0.241		
Coexisting conditions				
Diabetes	1.637 (0.295-9.076)	0.573		
Hypertension	0.977 (0.112-8.492)	0.983		
Preoperative RRT	6.567 (0.711-60.614)	0.097		
Perioperative factors				
Recipient operation time (min)	0.999 (0.990-1.008)	0.865		

Intraoperative RBC (units)	1.001 (0.898-1.116)	0.649		
Postoperative factor				
APACHE IV-LT specific predicted mortality*	1.075 (1.033-1.118)	<0.001	1.101 (1.026-1.181)	0.007
Inotropic support on admission to ICU	8.822 (0.933-83.401)	0.057	28.763 (1.140-725.462)	0.041
Postoperative AKI	17.805 (3.163-100.211)	0.001		
Postoperative RRT	38.600 (6.668-223.438)	<0.001	90.079 (4.635-1750.768)	0.003
Biliary complication	1.932 (0.220-16.966)	0.553		
Reoperation	1.561 (0.178-13.655)	0.687		
Postoperative RBC (units)	1.056 (0.990-1.125)	0.007		
Surgical site infection	5.213 (0.573-47.433)	0.143		
ICU readmission	21.556 (4.063-114.349)	<0.001	54.833 (2.794-1076.078)	0.008
Preoperative hospital LOS	1.060 (1.018-1.104)	0.004		

P value of Hosmer-Lemeshow goodness-of –fit test of multivariable analysis: 1.000. Nigelkerke R^2: 0.662. *APACHE IV-LT specific predicted mortality which has the highest AUC was chosen as the representative variable among other scoring systems for multivariable analysis.

†After adjusting for HCV LC, preoperative RRT, vasopressors on admission, postoperative RBC transfusion, surgical site infection, and preoperative hospital LOS.

LC, liver cirrhosis; MELD, model for end-stage liver disease; MELD-Na, model for end-stage liver disease-Na; APACHE, acute physiology and chronic health evaluation; SAPS, Simplified Acute Physiology Score; CTP, Child-Turcotte-Pugh score; AKI, acute kidney injury; RRT, renal replacement therapy; ICU , intensive care unit; LOS, length of stay.

Supplementary Table 3 Factors associated with in-hospital mortality after deceased donor liver transplantation

Variables	Unadjusted OR (95% CI)	*P* value in univariable-analysis	Adjusted OR† (95% CI)	*P* value in multivariable - analysis
Age	1.021 (0.965-1.079)	0.476		
Gender (female)	1.427 (0.402-5.058)	0.582		
Body mass index (kg/m^2)	1.070 (0.927-1.235)	0.356		
Initial Diagnosis				
Hepatocellular carcinoma	1.019 (0.287-3.623)	0.977		
Liver cirrhosis				
Hepatitis B virus LC	0.955 (0.280-3.252)	0.941		
Hepatitis C virus LC	0.828 (0.100-6.848)	0.861		
Alcoholic LC	0.869 (0.179-4.211)	0.862		
Others	1.013 (0.121-8.467)	0.990		
Preoperative corrected sodium	1.119 (1.019-1.229)	0.019	1.169 (1.012-1.350)	0.034
MELD score	1.095 (1.030-1.164)	0.003		
Coexisting conditions				
Diabetes	0.729 (0.151-3.518)	0.694		
Hypertension	1.103 (0.226-5.383)	0.903		

Chronic kidney disease	1.013 (0.121-8.467)	0.990		
Preoperative RRT	5.079 (0.920-28.053)	0.062		
Perioperative factors				
Recipient operation time (min)	1.003 (0.996-1.009)	0.475		
Intraoperative RBC (units)	0.986 (0.914-1.063)	0.708		
Postoperative factor				
APACHE IV-LT specific predicted mortality*	1.068 (1.027-1.110)	0.001	1.051 (1.009-1.094)	0.016
Inotropic support on admission to ICU	3.766 (1.018-13.927)	0.047		
Postoperative AKI	16.375 (3.387-79.178)	0.001		
Postoperative RRT	37.250 (7.458-186.045)	<0.001	24.241 (3.716-158.131)	0.001
Biliary complication	0.882 (0.106-7.321)	0.908		
Reoperation	2.921 (0.713-11.967)	0.136		
Postoperative RBC (units)	1.030 (0.996-1.065)	0.085		
Surgical site infection	9.048 (2.449-33.431)	0.001		
ICU readmission	5.887 (1.350-25.679)	0.018		
Preoperative hospital LOS	1.016 (0.979-1.056)	0.401		

P value of Hosmer-Lemeshow goodness-of –fit test of multivariable analysis: 0.995. Nigelkerke R^2: 0.570.

* APACHE IV-LT specific predicted mortality showed the highest AUC and was chosen as the representative scoring system for multivariable analysis.

[†]After adjusting for MELD score, preoperative RRT, inotropic support on admission to ICU, postoperative AKI, reoperation, surgical site infection, and ICU readmission.

LC, liver cirrhosis; MELD, model for end-stage liver disease; MELD-Na, model for end-stage liver disease-Na; APACHE, acute physiology and chronic health evaluation; SAPS, Simplified Acute Physiology Score; CTP, Child-Turcotte-Pugh score; AKI, acute kidney injury; RRT, renal replacement therapy; ICU , intensive care unit; LOS, length of stay.

Supplementary Table 4 Performance of APACHE IV, SAPS 3, APACHE II, MELD-Na, MELD, and CTP models on prediction of 3-month mortality

	APACHE IV - LT specific predicted mortality	SAPS 3	APACHE II	MELD-Na	MELD	CTP
AUC (95% CI)	0.87 (0.79-0.95)	0.71 (0.55-0.97)*	0.73 (0.55-0.91)	0.73 (0.56-0.89)	0.76 (0.60-0.92)	0.66 (0.52-0.81)
Cutoff point	44	55	24	22	24	11
H-L C-test χ^2	9.46	6.02	4.49	8.64	7.61	5.00
p-value	0.31	0.65	0.81	0.37	0.47	0.66
H-L H-test χ^2	5.51	9.05	14.10	5.21	7.32	5.97
p-value	0.70	0.34	0.08	0.73	0.50	0.65
SMR (95% CI)	NA	0.15 (0.09-0.23)	0.11 (0.06-0.17)	NA	NA	NA
Sensitivity	0.89	0.78	0.72	0.78	0.70	0.56
Specificity	0.77	0.70	0.86	0.67	0.79	0.75
PPV	0.11	0.08	0.14	0.07	0.10	0.07
NPV	1.00	0.99	0.99	0.99	0.99	0.98

Statistical comparison of APACHE IV-LT specific predicted mortality with *CTP scores ($p = 0.020$) after Holm adjustment for multiple comparisons.

APACHE, acute physiology and chronic health evaluation; SAPS, Simplified Acute Physiology Score; LT, liver transplantation, MELD, model for end-stage liver disease; MELD-Na, model for end-stage liver disease-Na; CTP, Child-Turcotte-Pugh score; AUC, area under the receiver operating curve; CI, confidence interval; PPV, positive predictive value; NPV, negative predictive value; H-L C-test. Hosmer-Lemeshow C-statistics; H-L H-test, Hosmer-Lemeshow H-statistics; NA, not applicable; SMR, Standardized mortality ratio.

Supplementary Table 5 Comparison of APACHE IV, SAPS 3, APACHE II, MELD-Na, and CTP scores according to MELD score in predicting 1-year mortality.

MELD score	Non-survivor /total patients	AUC (95% confidence interval)					
		APACHE IV -LT specific predicted mortality	SAPS 3	APACHE II	MELD-Na	MELD	CTP
< 15	7/309	0.85 (0.70-0.99)	0.79 (0.63-0.96)	0.71 (0.51-0.90)	0.50 (0.24-0.76)	-	0.57 (0.36-0.77)
15-24	7/161	0.73 (0.59-0.86)	0.44 (0.22-0.66)	0.45 (0.30-0.61)	0.56 (0.36-0.76)	-	0.54 (0.30-0.79)
≥25	11/120	0.79 (0.63-0.95)	0.71 (0.54-0.88)	0.82 (0.71-0.92)	0.60 (0.40-0.79)	-	0.55 (0.35-0.74)
Living donor	10/412	0.84 (0.76-0.93)	0.73 (0.55-0.92)	0.79 (0.66-0.93)	0.56 (0.34-0.77)	0.53 (0.31-0.75)	0.59 (0.41-0.77)
Deceased donor	15/178	0.76 (0.73-0.79)	0.58 (0.41-0.75)*	0.58 (0.39-0.76)†	0.62 (0.46-0.78)‡	0.64 (0.48-0.80)	0.56 (0.39-0.73)
All	25/590	0.83 (0.76-0.90)	0.71 (0.59-0.82)	0.73 (0.63-0.83)	0.67 (0.55-0.79)	0.69 (0.57-0.80)	0.64 (0.53-0.75)

Statistical comparison of APACHE IV-LT specific predicted mortality in deceased donor liver transplantation with *SAPS 3 scores (*P* < 0.001), †APACHE Ⅱ scores (*P* < 0.001), and ‡MELD-Na scores (*P* = 0.002) after Holm adjustment for multiple comparisons.

APACHE, acute physiology and chronic health evaluation; LT, liver transplantation; MELD, model for end-stage liver disease; MELD-Na, model for end-stage liver disease-Na; SAPS, Simplified Acute Physiology Score; CTP, Child-Turcotte-Pugh score

Supplementary Table 6 Comparison of patient characteristics between 1-year survivors and non-survivors

Variables	In-hospital survivor (n = 565)	In-hospital non-survivor (n = 25)	*p*- value
Age (years)	54 [49-60]	55 [46-68]	0.204*
Sex (M/F)	104 (71.0)/164 (29.0)	17 (68.0)/8 (32.0)	0.822
Body mass index (kg/m^2)	23.5 [21.4-26.1]	24.7 [22.7-27.1]	0.093*
Initial Diagnosis			
Hepatocellular carcinoma	314 (55.6)	11 (44.0)	0.306
Liver cirrhosis			
Hepatitis B virus liver cirrhosis	375 (66.4)	13 (52.0)	0.195
Hepatitis C virus liver cirrhosis	55 (9.7)	5 (20.0)	0.164
Alcoholic liver cirrhosis	75 (13.3)	4 (16.0)	0.762
Others	34 (6.0)	2 (8.0)	0.660
Preoperative sodium (mmol/L)	137 [131-140]	136 [132-142]	0.256*
Preoperative corrected sodium (mmol/L)	137 [132-140]	139 [132-142]	0.205*
MELD score	13 [9-22]	24 [14-36]	0.002*
MELD-Na score	16 [10-27]	26 [17-36]	0.004*
CTP score	8 [6-10]	10 [8-12]	0.015*
Coexisting conditions			
Diabetes mellitus	130 (23.0)	8 (32.0)	0.333

Hypertension	94 (16.6)	6 (24.0)	0.410
Chronic kidney disease	28 (5.0)	1 (4.0)	1.000
Preoperative RRT	18 (3.2)	4 (16.0)	0.011
Perioperative factors			
Donor status			0.002
Living / Deceased	402 (71.2)/163 (28.8)	10 (40.0)/15 (60.0)	
Operation type			0.001
Elective/ Emergency	392 (69.4)/173 (30.6)	9 (36.0)/16 (64.0)	
Operation time (min)	385 [320-445]	350 [300-460]	0.404*
Intraoperative RBC (units)	4 [0-10]	8 [6-11]	0.007*
Postoperative factors			
APACHE IV score	64 [51-80]	86 [73-113]	<0.001*
APACHE IV-LT specific predicted mortality (%)	22.4 [10.6-41.8]	64.1 [41.8-87.3]	<0.001*
SAPS 3 score	46 [38-56]	58 [47-72]	<0.001*
SAPS 3 predicted mortality (%)	12.0 [5.0-28.0]	32 [13-59]	<0.001*
APACHE II score	16 [13-20]	24 [17-32]	<0.001*
APACHE II predicted mortality (%)	14.3 [9.7-23.0]	34.8 [16.1-63.2]	<0.001*
APACHE II predicted mortality- LT specific diagnostic weight (%)	9.5 [6.3-15.8]	25.2 [10.8-52.0]	<0.001*
Inotropic support on admission to ICU	30 (5.3)	6 (24.0)	0.003
Mechanical ventilation duration (hours)	6.5 [4.0-13.3]	21.0 [7.0-156.5]	<0.001*
Postoperative AKI	75 (13.3)	15 (60.0)	<0.001

Postoperative RRT			
Biliary complications	54 (9.6)	3 (12.0)	0.725
Reoperation	62 (11.0)	7 (28.0)	0.019
Postoperative RBC (units)	0 [0-2]	6 [0-14]	<0.001*
Surgical site infection	27 (4.8)	8 (32.0)	<0.001
ICU readmission	26 (4.6)	8 (32.0)	<0.001
Postoperative ICU LOS (days)	4.1 [3.6-5.5]	7.2 [4.9-29.9]	<0.001*
Hospital LOS (days)	21 [15-36]	43 [24-75]	0.002*
Preoperative hospital LOS (days)	4 [3-13]	13 [3-26]	0.016*
Postoperative hospital LOS (days)	14 [11-20]	25 [9-41]	0.190*

Data are expressed as median [interquartile range] or number (%). *Mann-Whitney U test.

MELD, model for end-stage liver disease; MELD-Na, model for end-stage liver disease-Na; APACHE, acute physiology and chronic health evaluation; LT, liver transplantation; SAPS, Simplified Acute Physiology Score; CTP, Child-Turcotte-Pugh score; AKI, acute kidney injury; RRT, renal replacement therapy; ICU , intensive care unit; LOS, length of stay.

Supplementary Table 7 Survival rate after liver transplantation according to MELD scores.

MELD score	No. of patients	Survival (%)			
		1 month	6 months	12 months	18 months
< 15	309	99.4	99.4	97.7	96.4
15-24	161	98.1	98.1	95.7	95.7
≥25	120	94.2	90.8	90.8	90.0
All	590	98.0	97.3	95.8	94.9
p value*		0.003	<0.001	0.006	0.022

* Comparison between groups with MELD scores <15 and ≥25

Supplementary Table 8. Survival rate after liver transplantation according to APACHE IV scores.

APACHE IV score	No. of patients	Survival (%)			
		1 month	6 months	12 months	18 months
≤ 40	59	100.0	100.0	100.0	98.3
41-60	183	99.5	99.5	98.9	98.9
61-80	206	99.5	99.0	97.1	95.1
81-100	101	94.1	93.1	90.1	90.1
≥101	41	90.2	85.4	82.9	82.9
All	590	98.0	97.3	95.8	94.9
p value*		0.001	<0.001	<0.001	0.003
p value†		<0.001	<0.001	<0.001	0.022

* Comparison between groups with APACHE IV scores ≤40 and 81- 100.

† Comparison between groups with APACHE IV scores ≤40 and ≥101
